# A2A-D2 Heteromers on Striatal Astrocytes: Biochemical and Biophysical Evidence

**DOI:** 10.3390/ijms20102457

**Published:** 2019-05-17

**Authors:** Simone Pelassa, Diego Guidolin, Arianna Venturini, Monica Averna, Giulia Frumento, Letizia Campanini, Rosa Bernardi, Pietro Cortelli, Giovanna Calandra Buonaura, Guido Maura, Luigi F. Agnati, Chiara Cervetto, Manuela Marcoli

**Affiliations:** 1 Department of Pharmacy, Section of Pharmacology and Toxicology, University of Genova, Viale Cembrano 4, 16148 Genova, Italy; pelassa@difar.unige.it (S.P.); a.venturini@tigem.it (A.V.); frumento@difar.unige.it (G.F.); maura@difar.unige.it (G.M.); marcoli@pharmatox.unige.it (M.M.); 2 Department of Neuroscience, University of Padova, Via Gabelli 63, 35122 Padova, Italy; diego.guidolin@unipd.it; 3 Department of Experimental Medicine, Section of Biochemistry, University of Genova, Viale Benedetto XV, 1, 16132 Genova, Italy; monica.averna@unige.it; 4 Division of Experimental Oncology, San Raffaele Scientific Institute, Via Olgettina 60, 20132 Milano, Italy; Letizia.campanini@gmail.com (L.C.); bernardi.rosa@hsr.it (R.B.); 5 Department of Biomedical and NeuroMotor Sciences (DIBINEM) Alma Mater Studiorum-University of Bologna, Via Altura 3, 40139 Bologna, Italy; pietro.cortelli@unibo.it (P.C.); giovanna.calandra@unibo.it (G.C.B.); 6 IRCCS Istituto delle Scienze Neurologiche di Bologna, Via Altura 3, 40139 Bologna, Italy; 7 Department of Diagnostic, Clinical Medicine and Public Health, University of Modena and Reggio Emilia, Via Campi 287, 41125 Modena, Italy; luigi.agnati@gmail.com; 8 Department of Neuroscience, Karolinska Institutet, Retzius väg 8, 171 65 Stockholm, Sweden; 9 Centre of Excellence for Biomedical Research CEBR, University of Genova, Viale Benedetto XV, 5, 16132 Genova, Italy

**Keywords:** A2A-D2 heteromers, co-immunoprecipitation, proximity ligation assay, rat striatum, striatal astrocyte processes, striatal slices

## Abstract

Our previous findings indicate that A2A and D2 receptors are co-expressed on adult rat striatal astrocytes and on the astrocyte processes, and that A2A-D2 receptor–receptor interaction can control the release of glutamate from the processes. Functional evidence suggests that the receptor–receptor interaction was based on heteromerization of native A2A and D2 receptors at the plasma membrane of striatal astrocyte processes. We here provide biochemical and biophysical evidence confirming that receptor–receptor interaction between A2A and D2 receptors at the astrocyte plasma membrane is based on A2A-D2 heteromerization. To our knowledge, this is the first direct demonstration of the ability of native A2A and D2 receptors to heteromerize on glial cells. As striatal astrocytes are recognized to be involved in Parkinson’s pathophysiology, the findings that adenosine A2A and dopamine D2 receptors can form A2A-D2 heteromers on the astrocytes in the striatum (and that these heteromers can play roles in the control of the striatal glutamatergic transmission) may shed light on the molecular mechanisms involved in the pathogenesis of the disease.

## 1. Introduction

It has been suggested that receptor–receptor interactions (RRI) play roles in the information handled at the membrane level in the central nervous system (CNS). The first evidence for structural RRIs between G protein-coupled receptor (GPCR) was provided in the 1980s [1,2,3,4,5]. The existence of GPCR complexes at the neuronal membrane level was then supported by several groups, and the development of biophysical techniques to detect spatial proximity of protein molecules contributed to build up evidence for GPCR complexes [6,7,8,9]. It has been suggested that GPCR complexes are potentially involved in neurodegeneration and neuroinflammation [10], and in learning and memory processes [11,12], since their formation can modulate the synaptic weight [13]. GPCR complexes represent horizontal molecular networks (specialized plasma membrane micro-circuits) that can function as “intelligent interfaces” between the extracellular and the intracellular environment [14,15,16,17,18]. Notably, A2A-D2 heteromers emerging from the interaction of the adenosine A2A receptor and the dopamine D2 receptor in the striatal neuron plasma membrane opened up new comprehension of the Parkinson’s disease (PD) pathophysiology and of the adverse anti-Parkinson’s drug reaction [8,19,20,21,22,23,24,25,26,27].

Despite increasing recognition of the astrocyte involvement in neurodegeneration processes and in the CNS vulnerability to diseases, the existence of GPCR RRIs at the astrocytic plasma membrane has been barely investigated. 

We recently reported that A2A and D2 receptors are expressed in adult rat striatal astrocyte processes and their interaction controls glutamate release at the astrocyte level; functional evidence strongly suggested that the A2A-D2 RRI was based on receptor heteromerization [28,29]. As a matter of fact, definitive proof for receptor heteromerization rests on biochemical and biophysical evidence for structural interaction between the receptors and spatial proximity of protein molecules. Here, by co-immunoprecipitation and proximity ligation assay (PLA) approaches we provide both biochemical and biophysical evidence for the ability of native striatal astrocytic A2A and D2 receptors to heteromerize. 

Altogether the functional, biochemical and biophysical data on striatal astrocytic A2A-D2 heteromers indicate new aspects of the regulation of the dopaminergic control of glutamatergic transmission in the striatum. As astrocytes have been proposed to participate in striatal glutamatergic dysfunction in PD [30,31,32], our findings could open further investigations into possible pathogenic disease mechanisms and open possibilities for the development of new drugs targeting the disease.

## 2. Results

### 2.1. D2 and A2A Receptors Expressed on the Striatal Astrocytic Process Membrane Physically Interact 

**Figure 1:**
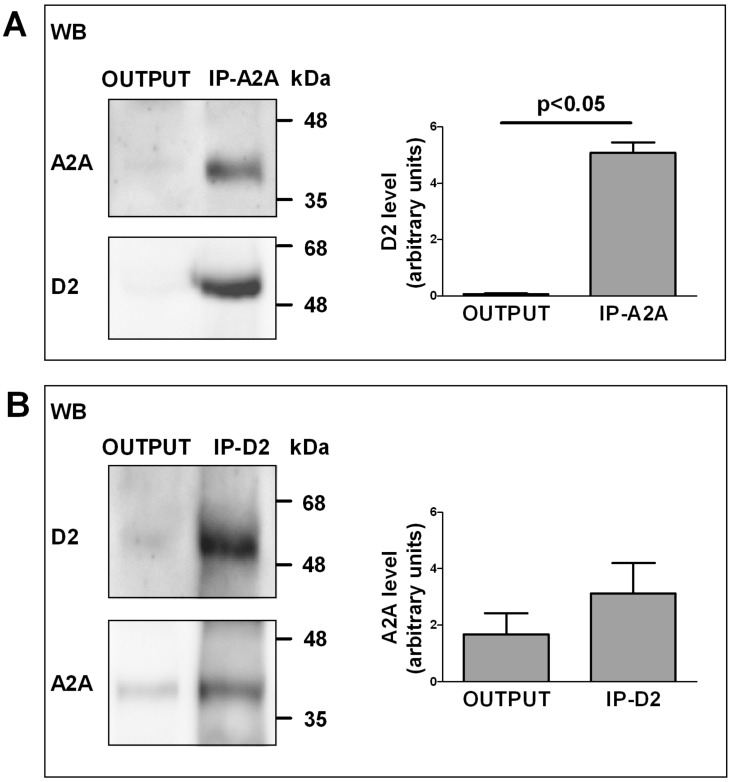
Interaction between A2A and D2 on rat striatal astrocyte processes: co-immunoprecipitation. (**A**) Aliquots (300 µg) of Triton X-100-soluble proteins obtained from gliosomes were immunoprecipitated with 1 µg of anti-A2A antibody as described in Methods. Immunoprecipitated (IP) and not immunoprecipitated (Output) materials were analyzed by immunoblotting using the anti-A2A antibody. IP and Output were also analyzed using anti-D2 antibody. A representative blot (of three) is shown. D2 immunoreactive bands were quantified and the data were reported in the graph. Values are means ± SEM (*n* = 3). (**B**) Aliquots (300 µg) of Triton X-100-soluble proteins obtained from gliosomes were immunoprecipitated with 1 µg of anti-D2 antibody as described in Methods. IP and Output were analyzed by immunoblotting using the anti-D2 antibody. IP and Output were also analyzed using anti-A2A antibody. A representative blot (of three) is shown. A2A immunoreactive bands were quantified and the data were reported in the graph. Values are means ± SEM (*n* = 3).

The capability of A2A and D2 receptors on striatal astrocytes to physically interact was investigated by co-immunoprecipitation assay on purified striatal astrocyte processes. By co-immunoprecipitation, we found that the A2A and the D2 receptors expressed on the striatal astrocytic processes physically interact. In particular, as shown in Figure 1, by analyzing immunoprecipitated and output material by immunoblotting using the anti-D2 and the anti-A2A antibodies whose high specificity was previously verified [33,34,35,36,37,38], we found that the D2 receptor almost completely immunoprecipitated together with the A2A receptor. Conversely, only a fraction of the A2A receptor was found to immunoprecipitate together with the D2 receptor. The findings indicate that the D2 receptor expressed on the striatal astrocytic process is completely associated with the A2A receptor, while the A2A receptor can be also found not associated with the D2 receptor.

### 2.2. D2 and A2A Receptors Expressed on Striatal Astrocytes Can Form Heteromers 

**Figure 2:**
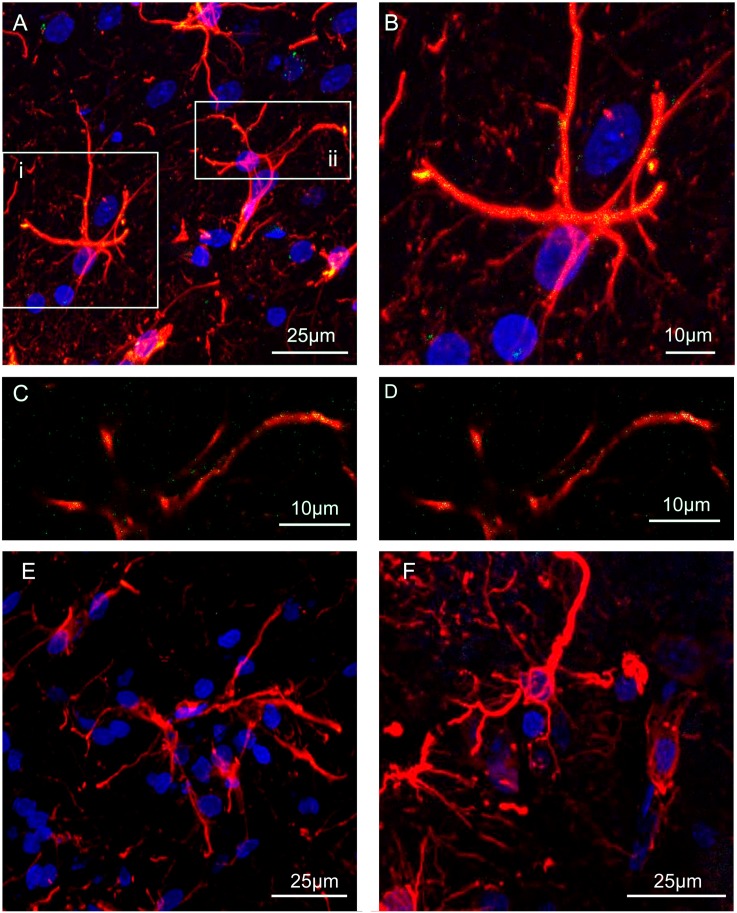
A2A-D2 heterodimers on rat striatal astrocytes: proximity ligation assay. Detection of in situ PLA A2A-D2 heteroreceptor complexes was carried out with primary antibodies (rabbit polyclonal anti-A2AR, mouse monoclonal anti-D2R and goat polyclonal anti-GFAP) in rat striatal slices. (**A**) The merge of the maximum intensity projections of a representative field (240x240 µm; z 10 µm) is shown; GFAP (red), DAPI (blue), PLA for A2A-D2 heteroreceptor complexes appears as yellow clusters. The boxed region (i) is shown at a higher magnification in (**B**). (**C**) Merged confocal images of a single z stack of the boxed region (ii) at a higher magnification: double immunolabeling for GFAP (red) and PLA A2A-D2 heteroreceptor complexes (green). (**D**) Colocalized map of the boxed region (ii); the colocalization analysis plugins—colocalization threshold with GFAP as ROI—was used to create the colocalized map; ImageJ Fiji software. A complete lack of stain for PLA A2A-D2 heteroreceptor complexes was obtained in the negative control experiments, performed avoiding the conjugation of a primary antibody with the Duolink Probes. In the figure the merges of the maximum intensity projections of two representative fields are shown: PLA for A2A-D2. heteroreceptor complexes without the primary anti-A2A antibody (**E**) or the primary anti-D2 antibody (**F**). Scale bar 25 µm or 10 µm

As illustrated in Figure 2 astrocytes were identified in striatal slices by the astrocyte marker glial fibrillary acidic protein (GFAP). The presence of A2A and D2 receptors on striatal astrocytes and their ability to heteromerize was assessed by PLA (Figure 2A–D). The in situ PLA assay showed green spots for A2A-D2 heteroreceptor complexes in GFAP-positive astrocytes. Samples in which a single primary antibody was administered were used as negative controls and, as expected, they did not exhibit any staining (see Figure 2E,F). Altogether these data support the existence of A2A-D2 heteromers in the rat striatum.

## 3. Discussion

We have previously reported that the adenosine A2A receptor and the dopamine D2 receptor are expressed on striatal astrocytes from adult rats; the finding that both the receptors were co-expressed on GFAP-positive astrocytic structures [29] indicated that native A2A and D2 receptors on striatal astrocytes might interact through A2A-D2 RRI. In particular, the A2A and D2 receptors appeared co-expressed together with the vesicular glutamate transporter VGLUT1 on a subpopulation of processes, and were able to functionally interact to control the glutamate release from the processes. In fact, the D2 receptor activation reduced the release of glutamate from the processes, while the activation of the A2A receptor, which was per se ineffective on the glutamate release [28], counteracted the effect of D2 receptor activation. Notably, the synthetic peptide VLRRRRKRVN corresponding to the D2 receptor region involved in electrostatic interaction underlying A2A-D2 receptor heteromerization [39] disrupted the A2A effect [28], further supporting a key role for the A2A-D2 RRI and suggesting that the RRI involves electrostatic interaction between the D2 receptor third intracellular loop and the A2A receptor tail. It was also found that homocysteine, a known allosteric ligand of the A2A-D2 heteroreceptor complex [40], can increase the inhibitory effect of the A2A receptor on the vesicular glutamate release induced by D2 receptor activation. Altogether these pharmacological findings suggested that the A2A-D2 RRI was based on receptor heteromerization [28,29] also indicating that the nature and mechanism of interaction of native A2A and D2 receptors on striatal astrocyte processes plasma membrane are similar to those of co-transfected receptors [39,41].

However, only a combination of multiple biochemical and structural data can provide a direct demonstration of receptor heteromers [42]. In this respect, the results of the present study might provide final evidence that native A2A receptor for adenosine, and D2 receptors for dopamine can form heteromers on the plasma membrane of striatal astrocytes. In fact, direct physical evidence of receptor–receptor interaction was provided by co-immunoprecipitation, and confirmed by in situ PLA, which selectively identify pairs of receptor molecules located at a distance lower than 16 nm from each other, a condition consistent with the physical interaction needed for receptor heterodimerization. 

### 3.1. Physical Interaction between Astrocytic Adenosine A2A and Dopamine D2 Receptor: Co-Immunoprecipitation

Although co-immunoprecipitation per se is not sufficient to establish the ability of receptors to heteromerize, as it does not exclude indirect physical interaction between the two receptors, receptors that form heteromers are expected to co-precipitate. Thus, the findings here reported on A2A and D2 receptors in astrocyte processes is consistent with the ability of the receptors to interact physically in receptor heteromers. They also add further support to the previously obtained functional evidence [28]. As a matter of fact, the A2A receptor was able to completely precipitate the D2 receptor, while when precipitating with the D2 receptor, a fraction of the A2A receptor was still found in the medium. The finding indicates that while the D2 receptor expressed on the plasma membrane of astrocyte processes is always coupled to A2A receptor, A2A receptor may also exist without being associated with D2 receptors. Interestingly, although homodimers seemed the functional form of the transfected A2A receptor on the cell plasma membrane, it was predicted that—due to the high presence of D2 in striatal neuron cultures—in striatal neurons A2A-D2 heteromers may co-exist with A2A-A2A homomers (see [43]). Indeed, investigation on striatal presynaptic nerve terminals would allow a better understanding of the role of astrocytic and neuronal RRIs in the interplay between dopamine and adenosine and in the control of glutamatergic transmission at striatal synapses. The co-immunoprecipitation finding on astrocyte processes is in good agreement with the functional data showing that the effect of activation of the D2 receptor (inhibition of depolarization-evoked release of glutamate [28]) was completely prevented by activation of the A2A receptor through a receptor–receptor interaction since it was destroyed by a peptide interfering with the receptor heteromerization [28]. 

### 3.2. Physical Interaction between Astrocytic Adenosine A2A and Dopamine D2 Receptor: Proximity Ligation Assay

PLA is considered a particularly important technique to demonstrate receptor heteromerization, because it allows the identification of native receptor molecules that are up to 16 nm apart, a distance considered crucial for heteromer formation [44,45]. The presence of PLA signal in GFAP-positive cells in striatal slices, therefore, is strongly indicative of the presence of heteromers made by native A2A and D2 receptors on the plasma membrane of astrocytes in the rat striatum and confirms that the functional interaction between the receptors, and the receptor co-immunoprecipitation, are based on receptor heteromerization. 

As a final remark, it must be observed that the data here reported represent, at least to our knowledge, the first direct demonstration that native G protein-coupled receptors can interact in functional heteromers to control gliotransmitter release from astrocytes. As a matter of fact, the presence of native G protein-coupled receptors heteromers on astrocytes has scarcely been studied, and only sporadic reports are available (heteromerization of the cannabinoid CB2 receptor and the non-cannabinoid receptor GPR55 in the astrocytes of prefrontal cortex of suicides [46]; heterodimeric sweet taste receptors, T1R2 and T1R3 [47]; heteromerization of the serotonin 5-HT1A and D2 receptors in astrocytes and neurons in the mouse prefrontal cortex [48]).

## 4. Materials and Methods 

### 4.1. Experimental Animals

Male Sprague Dawley adult rats (200–250 g) were housed at the animal care facility of the Department of Pharmacy (DIFAR), University of Genova, Italy, under a light–dark schedule (lights on 7 AM–7 PM). Constant temperature (22 ± 1°C) and relative humidity (50%) were maintained; the animals had free access to standard diet, and water ad libitum. The care of the animals and experimental procedures were approved by the University of Genova Ethical Committee (protocol number 301116 of 30 November 2016), complying with the Directive of 22 September 2010 (2010/63/EU) of the European Community’s Parliament and Council and with the Italian Legislative Decree n. 26/2014. Every effort was made to reduce the number of the animals used and to minimize the animal suffering.

### 4.2. Preparation of Striatal Slices 

After decapitation, the striatum was rapidly removed and processed to prepare cryostat sections, essentially as previously described [49]. Briefly, the striatum was frozen in liquid nitrogen and sliced on a cryostat (Frigocut 2008E, Leica Biosystems, Wetzlar, Germany). Coronal sections (10-µm thick) were collected on poly(l-lysine)-coated slides and stored at −80 °C until further processing. 

### 4.3. Proximity Ligation Assay

PLA was carried out essentially as described in [45]. In situ PLA was performed on 10µm rat striatal slices using the primary antibodies (mouse anti-A2A (1:200, Merck Millipore Corporation, 05-717, Burlington, MA, USA); rabbit anti-D2R (1:200, Alomone Labs, ADR-002, Jerusalem, Israel); goat anti-GFAP (1:500, Santa Cruz Biotechnoloy Inc, Dallas, TX, USA, sc-6170), the Duolink in situ PLA detection kit (DUO92014, Sigma-Aldrich, St Louis, MO, USA) and Alexa Fluor 546-conjugated donkey anti-goat (1:500; Molecular Probes, Eugene, OR, USA). PLA was performed according to the manufacturer’s instructions using the Duolink Detection Kit (DUO92014, DUO92001, DUO92005 Sigma-Aldrich). Briefly, slices were first washed twice for 10 min in PBS solution, then blocked with 3% normal serum and 0.2% Triton X-100 in phosphate-buffered saline (PBS) for 1 h in a humid chamber at room temperature. Subsequently slices were permeabilized in 0.2% Triton X-100 in PBS for 15 min, washed two times in PBS solution for 10 min, blocked with Blocking Solution in a humid chamber for 30 min at 37 °C and then incubated with the primary antibody (anti-A2A and anti-D2) in Antibody Diluent solution in a humid chamber overnight at 4 °C. Thereafter, the sections were rinsed with PBS solution and incubated with species-specific secondary antibodies conjugated to complementary oligonucleotides for 1 h at 37 °C (DUO92001, DUO92005 Sigma-Aldrich). After hybridization, ligation and amplification steps were performed using the manufacturer’s instruction and fluorescence images were acquired enabling the visualization of the A2A-D2 receptor heteromer by green fluorescent at confocal microscopy.

For GFAP colocalization analysis, after the amplification step the slices were rinsed in Wash Buffer A, incubated with goat anti-GFAP in Antibody Diluent solution in a humid chamber overnight at 4 °C and subsequently with Alexa Fluor 546-conjugated donkey anti-goat for 1 h at room temperature. After incubation in DAPI labeling solution, the sections were rinsed in PBS, mounted in a glycerol/PBS (1:1) solution and examined under a confocal laser scanning microscope (SP2 AOBS, Leica Microsystems, Mannheim, Germany). Negative control experiments were conducted avoiding the conjugation of the primary anti-A2A or anti-D2 antibody with the Duolink Probes and resulted in a complete lack of stain for PLA (see Figure 2E,F). The specificity of the double immunolabeling was verified by replacing the primary antibodies with PBS [49].

### 4.4. Preparation of Purified Astrocyte Processes

After decapitation, the striatum was rapidly removed and placed in ice-cold medium. Purified astrocyte processes (gliosomes) were prepared according to Nakamura’s method [50], as previously reported [28,29,51,52]. The result is a preparation of astrocytic processes with negligible neuronal contamination, containing gliotransmitter-loaded vesicles and capable of gliotransmitter secretion [28,29,51,52]. Briefly, the striatum was homogenized in 10 volumes of 10 mM Tris/HCl (pH 7.4 and containing 0.32 M sucrose) with a glass-Teflon tissue grinder (0.25 mm clearance). After initial centrifugation of the homogenate to remove nuclei and debris (5 min; 1000× *g*; 4 °C), the supernatant was stratified on a discontinuous Percoll gradient (2, 6, 10 and 20% (*v*/*v*) in Tris-buffered sucrose) and centrifuged (5 min; 33,500× *g*; 4 °C). Gliosomes were collected at the layer between 2% and 6% (*v*/*v*) Percoll and then washed by means of centrifugation. The confocal immunofluorescence analysis on striatal gliosomes indicated a negligible contamination of purified astrocyte processes by non-astrocytic fractions (less than 5%; see [28]). Notably, the striatal astrocyte processes, positive for the astrocytic markers GFAP or ezrin, expressed the vesicular glutamate transporter VGLUT1, and ultrastructural analysis demonstrated the presence of both smooth and clathrin-coated vesicles with a scattered distribution within the cytoplasm [28].

### 4.5. Immunoprecipitation and Immunoblot

Gliosomes obtained from the striatum of four animals were lysed in 50 mM sodium borate buffer, pH 7.5 with 1 mM EDTA and Protease Inhibitor Cocktail (lysis buffer) at 1mg/mL by three cycles of freezing and thawing followed by sonication. Protein quantification of lysate was performed using the Bradford method [53]. To perform the immunoprecipitation, gliosome lysate was centrifuged at 100,000× *g* for 30 min at 4°C. The pellet was washed once and then solubilized in 100 µL of 50 mM sodium borate, 0.1 mM EDTA, pH 7.5 (immunoprecipitation buffer) + 1% Triton X-100 at 37 °C for 1 h. Then 400 µL of immunoprecipitation buffer has been added to the lysate to dilute the Triton X-100 to 0.2% (Total of the membranes) and then centrifuged at 18,000× *g* for 15 min at 4°C. The supernatant has been precleared with protein G-sepharose and then incubated in the presence of 1 µg of anti-A2A-receptor antibody or anti-D2-receptor antibody at 4°C, overnight. Protein G-sepharose was then added to the sample and incubated for an additional 1 h at room temperature. The immunocomplexes were centrifuged at 400× *g* and aliquots of supernatant were submitted to SDS-PAGE. The immunocomplexes were washed three times with immunoprecipitation buffer + 0.1% Triton X-100, heated in SDS-PAGE loading buffer for 5 min and submitted to 10% SDS-PAGE followed by electroblotting onto a nitrocellulose membrane and saturated with phosphate-buffered saline, pH 7.5, containing 5% skim milk powder. The blots were probed with specific antibodies and the immunoreactive material was detected with a Bio-Rad Chemi Doc Extra Resolution and Sensitivity apparatus. Immunoreactive bands were quantified using the Quantity One 4.6.1 software (Bio-Rad, Hercules, CA, USA).

### 4.6. Calculations and Statistical Analysis

Means ± SEM of the number *n* of experiments are shown. Significance of the differences was analyzed by the non-parametric Mann-Whitney test, taking *p* < 0.05 to indicate statistical significance.

### 4.7. Materials

Triton^®^ X-100 was purchased from Sigma. Protein G-sepharose 4 Fast Flow, nitrocellulose membrane and ECL SELECT^®^ Detection System were obtained from GE Healthcare. Mouse anti-A2A primary antibody was purchased from Merck Millipore Corporation (05-717; [45]); goat anti-GFAP primary antibody was purchased from Santa Cruz Biotechnoloy Inc; rabbit anti-A2A and anti-D2 primary antibodies were purchased from Alomone Labs. The anti-rabbit secondary antibody and the Protease Inhibitor Cocktail were obtained from Cell Signaling Technology (Leiden, The Netherlands).

## 5. Conclusions

In conclusion, our data may provide definite demonstration of the ability of A2A and D2 receptors to form heteromers on the plasma membrane of glial cells. Specifically, knowledge of the ability to form A2A-D2 heteromers on striatal astrocytes gives new insights into the regulation of the dopamine control of glutamatergic transmission in the striatum. Interestingly, a similar regulatory mechanism based on A2A-D2 heteromers is also present in neurons [45]. Defining similarities and differences between neuronal and astrocytic A2A-D2 heteromers—e.g., at variance with astrocyte processes, activation of A2A receptor evoked glutamate release from striatal glutamatergic nerve terminals [28]—would require further investigation, to a better understanding of the role that striatal A2A-D2 heteromers might play as integrative input units in the control of striatal glutamatergic transmission. In any case, these new astrocyte-related aspects of glutamatergic transmission regulation in astrocyte-neuron intercellular communications in striatum might suggest new lines of research focusing on pathogenic mechanisms of PD and neuropsychiatric disorders (such as schizophrenia) and on the development of new pharmacological strategies for treatment. Notably, morphological changes with the expansion of perisynaptic astrocyte processes and with increase of the coverage of the striatal glutamatergic synapses by the astrocyte processes have been reported in PD models, and altered neuron-astrocyte interactions at glutamatergic synapses together with increased glutamate levels in the striatum [30,54] have been suggested to be involved in the PD pathophysiology. In fact, evidence is accumulating suggesting that dysfunction of astrocytes, and altered intercellular communication between astrocytes and neurons at the striatal synapses, can play an initiating role in pathophysiology of the PD [32]. A2A inhibition of astrocytic D2 signaling actually might contribute to striatal glutamatergic transmission dysfunction by increasing the extracellular glutamate levels [28,29]. Therefore, astrocytic A2A-D2 heteromers may represent a new pharmacological target for bivalent compounds (A2A antagonist-D2 agonist; see [21]) to control striatal glutamate transmission in PD. Although specificity of the pharmacological intervention would rest on selective targeting the striatal astrocytic vs neuronal A2A-D2 heteromers, blockade of the astrocytic A2A receptor might be predicted to have a greater impact on glutamate transmission regulation, when one considers the high expression of A2A receptors in PD [55,56], the colocalization of ectonucleotidases—producing adenosine from extracellular ATP—with the striatal astrocytic A2A receptors [57], and the expansion of the striatal perisynaptic processes in PD. In fact, investigation on the functioning of astrocytic A2A-D2 heteromers in the PD would get light on striatal D2 signaling, A2A signaling, and glutamate transmission in the disease.
